# The Deep Evolutionary Relationships of the Morphologically Heterogeneous Nolinoideae (Asparagaceae) Revealed by Transcriptome Data

**DOI:** 10.3389/fpls.2020.584981

**Published:** 2021-01-14

**Authors:** Ran Meng, Li-Ying Luo, Ji-Yuan Zhang, Dai-Gui Zhang, Ze-Long Nie, Ying Meng

**Affiliations:** Key Laboratory of Plant Resources Conservation and Utilization, College of Biological Resources and Environmental Sciences, Jishou University, Jishou, China

**Keywords:** Asparagaceae, Nolinoideae, transcriptome, deep relationship, phylogeny

## Abstract

The subfamily Nolinoideae of Asparagaceae is an extremely morphologically heterogeneous group, which is comprised of seven lineages, formerly known as Eriospermaceae, Polygonateae, Ophiopogoneae, Convallarieae, Ruscaceae s.s., Dracaenaceae, and Nolinaceae from different families or even orders. Their drastically divergent morphologies and low level of molecular resolution have hindered our understanding on their evolutionary history. To resolve reliable and clear phylogenetic relationships of the Nolinoideae, a phylogenetic study was conducted based on transcriptomic sequencing of 15 species representing all the seven lineages. A dataset containing up to 2,850,331 sites across 2,126 genes was analyzed using both concatenated and coalescent methods. Except for *Eriospermum* as outgroup, the transcriptomic data strongly resolved the remaining six lineages into two groups, one is a paraphyletic grade including the woody lineages of dracaenoids, ruscoids, and nolinoids and a monophyletic herbaceous clade. Within the herbaceous group, the Ophiopogoneae + *Theropogon* is sister to a clade that is composed of Convallarieae and the monophyletic Polygonateae. Our work provides a first robust deep relationship of the highly heterogeneous Nolinoideae and paves the way for further investigations of its complex evolution.

## Introduction

Phylogenetic studies have greatly changed our understanding on plant classification and evolution. To accord with molecular phylogenetic results, for example, Asparagales has been recircumscribed as the largest order in the monocotyledons with seven redefined or newly erected families (Chase et al., 1995; Fay et al., 2000; Seberg et al., 2012; Chen et al., 2013). Among them, Asparagaceae s.l. is greatly expanded as a highly complicated group with little shared characters, including seven subfamilies, i.e., Agavoideae, Aphyllanthoideae, Asparagoideae, Brodiaeoideae, Lomandroideae, Nolinoideae, and Scilloideae (Chase et al., 2009). Nolinoideae, formerly known as Ruscaceae s.l. or Convallariaceae s.l., is also a complex group comprised of seven heterogeneous lineages, traditionally known as Eriospermaceae, Polygonateae, Ophiopogoneae, Convallarieae, Ruscaceae s.s., Dracaenaceae, and Nolinaceae, and many of them have been traditionally placed in different families or even orders (Chase et al., 2009; Kim et al., 2010; APGIV, 2016). To avoid taxonomic confusions, Ruscaceae s.s., Dracaenaceae, and Nolinaceae are referred to as the ruscoids, the dracaenoids, and the nolinoids following Rudall et al. (2000) in this study, respectively.

The seven lineages in the Nolinoideae are extremely diverse in morphology, four of which are herbaceous and three are woody-like (Rudall et al., 2000; Yamashita and Tamura, 2000b; Kim et al., 2010). The first includes only *Eriospermum* Jacq. ex Willd. with about 80 species occurring mostly in southern Africa, which are perennial herbs with dormant structures of tubers, free perianth parts, and the epidermal hairs of the seed, traditionally assigned to its own monotypic family (Dahlgren et al., 1985). The APGIII (2009) system treated *Eriospermum* as a sister to the remainder in the Nolinoideae. The other three herbaceous groups (i.e., Polygonateae, Ophiopogoneae, and Convallarieae) had been well-known as Convallariaceae s.s. together separated from traditional Liliaceae (Rudall et al., 2000). They are usually rhizomatous and perennial herbs that occurred in the Northern Hemisphere and especially abundant in eastern and southeastern Asia (Dahlgren et al., 1985; Rudall et al., 2000).

Polygonateae is usually recognized into two lineages, one is the axillary-flowered group, including *Polygonatum* Mill., *Disporopsis* Hance and *Heteropolygonatum* M.N. Tamura & Ogisu, and the other is the terminal-flowered of *Maianthemum* F.H. Wigg. (Meng et al., 2008, 2014). Many published multi-locus analyses have demonstrated the monophyly of the tribe and its inclusion within Nolinoideae (Rudall et al., 2000; Meng et al., 2014), but there are still many controversies, especially the position of *Maianthemum* lineage (Kim et al., 2010; Floden and Schilling, 2018). The Ophiopogoneae includes *Ophiopogon* Meisn., *Liriope* Lour., and *Peliosanthes* Andrews, usually characterized with a sympodial rhizome, fruits dehisced at an early stage, seeds with sarcotesta, and basic chromosome number of *x* = 18 (Dahlgren et al., 1985). Molecular phylogenetic analyses strongly supported the monophyly of Ophiopogoneae within Nolinoideae (Kim et al., 2010; Wang et al., 2014; Wang and Yang, 2018). The tribe Convallarieae was expanded to include Aspidistreae (Conran and Tamura, 1998; Yamashita and Tamura, 2000b; Kim et al., 2010). They can be distinguished from the other herbaceous taxa by a short stem with monopodial rhizome and usually berries but rarely drupes (*Tricalistra* Ridl.) and usually basic chromosome numbers of *x* = 19 but rarely *x* = 18 (some *Aspidistra* Ker Gawl. spp.) (Yamashita and Tamura, 2004).

The woody taxa includes the ruscoids, the dracaenoids, and the nolinoids, usually recognized as different families. The dracaenoids include the genera of *Dracaena* L. and *Sansevieria* Thunb. mainly from tropical and subtropical Asia and Africa, which share the synapomorphic characters of berries, have no oils in guard cells, and have mucilage-filled cells with crystal raphides in their vegetative parts (Lu and Morden, 2014; Takawira-Nyenya et al., 2018). The ruscoids are comprised of three small genera from the Mediterranean and West Asia (*Ruscus* L., *Danae* Medik., and *Semele* Kunth) characterized by scale-like leaves, woody stems, berries, and a basic chromosome number of *x* = 20 (Rudall and Campbell, 1999). The nolinoids were previously represented as Nolinaceae including the four genera *Beaucarnea* Lem., *Calibanus* Rose, *Dasylirion* Zucc., and *Nolina* Michx. from the southern states of the United States through Mexico into Guatemala (Rojas-Piña et al., 2014). They were excluded from Agavaceae and placed close to Dracaenaceae, featured with arborescent, anomalously woody plants; terminal rosette leaves; and indehiscent fruits (Dahlgren et al., 1985).

The Nolinoideae is proved to be an extremely morphologically heterogeneous group with very few distinguishable synapomorphic characters from the other asparagoid members except for the absence of phytomelan in the seed coat (Rudall et al., 2000; Kim et al., 2010). The taxonomic classification in this group with drastically divergent morphologies has been problematic. Although molecular evidence indicated a well-supported group of the subfamily with the inclusion of the seven lineages as stated above, most internal branches among these seven groups have weak supports, except for *Eriospermum* that is strongly supported as a sister group to the remaining taxa (Rudall et al., 2000; Kim et al., 2010; Seberg et al., 2012). Low support values have also been observed in the other analyses and are the main argument for grouping all of them into one large family in the APG systems (Pires et al., 2006; Kim et al., 2010).

In a word, Nolinoideae is a well-supported group but without obvious synapomorphies and remains poorly resolved for its deep evolutionary relationships. However, it seems that traditional multi-locus approaches are unable to resolve a reliable and highly confident phylogenetic backbone of the subfamily. Rapid development of the next-generation sequencing technology has made large dataset accessible, allowing high-throughput selection of low or single-copy nuclear genes as phylogenetic markers (Wen et al., 2015a). *De novo* sequencing of transcriptome among many species has been tested recently as effective phylogenetic approaches (Wickett et al., 2011; Wen et al., 2015a; Ran et al., 2018). Coalescent analyses for species-tree estimation are becoming a dominant approach for reconstructing species histories over multi-locus data for recently diverged species. Recent studies used transcriptome datasets to successfully reconstruct phylogenies of various scales from various genera to angiosperm-wide or even land plants (Wen et al., 2013; Wickett et al., 2014; Zeng et al., 2017; Ran et al., 2018).

This study aims to reconstruct deep phylogenetic relationships of Nolinoideae and provide phylogenetic placement of some uncertain genera using both concatenated and coalescent analyses. Phylogenetic analyses were conducted based on transcriptomic data from representatives of the seven lineages. To avoid stochastic and systematic errors, we sequenced the transcriptomes of all sampled species and used OrthoFinder for orthology prediction, followed with multiple filter procedures.

## Materials and Methods

### Taxon Sampling and Transcriptome Sequencing

We provide transcriptomic sequencing from all seven lineages within Nolinoideae, including five species from the two lineages of Polygonateae, two from Convallarieae, one of Ophiopogoneae, two from the nolinoids, one of the ruscoids, two of the dracaenoids, and one from *Eriospermum* (Table 1). In addition, we sequenced *Theropogon pallidus* collected from southern Xizang of China, which is phylogenetically uncertain by traditional molecular data (Kim et al., 2010). *Eriospermum* was selected as outgroup as its sister relationship to the rest of the taxa of the subfamily is robustly supported by all previous studies (Fay et al., 2000; Jang and Pfosser, 2002; Kim et al., 2010; Wang et al., 2016). All samples for RNA sequencing were collected from the wild or botanical gardens and cultivated in a greenhouse of Jishou University (Table 1).

**TABLE 1:** Statistics of sampled transcriptomes.

Lineages	Species	Vouchers	No. of raw data	N50 (unigene)	No. of unigene
Polygonateae	*Polygonatum cyrtonema* Hua	Nie 5439	34,299,887	1,161	55,874
	*P. zanlanscianense* Pamp.	Nie 5440	31,928,107	1,184	57,181
	*P. sibiricum* Redouté	Nie 5450	23,507,559	1,145	49,732
	*Disporopsis aspera* (Hua) Engl.	Nie 5438	42,409,644	1,059	61,155
	*Maianthemum japonicum* (A. Gray) La Frankie	Nie 5451	25,027,168	1,078	52,653
Convallarieae	*Aspidistra fenghuangensis* K.Y. Lang	Nie 5442	30,779,512	1,131	57,501
	*Tupistra chinensis* Baker	Nie 5443	31,245,232	873	100,199
Ophiopogoneae	*Liriope platyphylla* F.T. Wang & Tang	Nie 5441	29,260,035	1,077	68,450
Nolinoids (Nolinaceae)	*Beaucarnea recurvata* Lem.	Nie 5446	23,863,397	915	100,217
	*Dasylirion longissimum* Lem.	Nie 5447	24,915,810	890	100,618
Ruscoids (Ruscaceae s.s.)	*Ruscus aculeatus* L.	Nie 5448	24,739,871	1,098	59,645
Dracaenoids (Dracaenaceae)	*Sansevieria trifasciata* Prain	Nie 5445	30,449,869	1,147	60,507
	*Dracaena angustifolia* Roxb.	Nie 5444	28,256,382	1,139	60,935
Eriospermaceae	*Eriospermum lancifolium* Jacq.	Nie 5449	23,675,661	1,177	55,412
–	*Theropogon pallidus* Maxim.	Nie 5452	21,598,854	1,144	59,484

*All specimens are deposited in Jishou University (JIU).*

Fresh juvenile leaf tissues of each sample were flash-frozen in liquid nitrogen. Total RNA was extracted using the RNA plant Plus Reagent (Tiangen, Beijing, China) and digested by DNase I (Promega, Madison, WI, United States). RNA concentration was measured using NanoDrop 2000 (Thermo), and integrity was assessed using the RNA Nano 6000 Assay Kit of the Agilent Bioanalyzer 2100 system (Agilent Technologies, CA, United States), following the manufacturer’s recommendations. The transcriptome library was constructed using NEBNext^®^Ultra^TM^ RNA Library Prep Kit for Illumina^®^ (NEB, United States) following the manufacturer’s protocols, and Illumina sequencing was performed by BioMarker (Beijing, China).

### Data Assembly and Identification of Orthologs

Raw reads were firstly checked with FastQC^1^ and trimmed using fastp with a quality filtering cutoff of 20 (Chen et al., 2018). The clean reads were *de novo* assembled with Trinity version 2.8.4 (Grabherr et al., 2011) using default parameters. Redundancy reduction was done for the raw assemblies, using CD-HIT with a threshold of 20 (Li and Godzik, 2006). For each transcriptome, transcripts were translated into peptides using default settings in TransDecoder version 0.36^2^. The quality and completeness of the final transcriptomes (unigene sets) for all samples were also benchmarked with BUSCO version 3.0.2 according to conserved ortholog content (Waterhouse et al., 2017). The analysis was carried out using the Liliopsida odb10 plant-specific reference database following default parameters of the software (Kriventseva et al., 2018) to obtain single-copy gene dataset with more strict criteria.

The conserved orthogroups (OG) were identified from the sets of translated proteins using OrthoFinder version 2.2.6 (Emms and Kelly, 2015) and further filtered using the following criteria: (i) sequences are missed in at least one species; (ii) the average copy number of an OG is greater than five; and (iii) the median copy number of an OG is greater than two. We used a rigorous comprehensive methodology for quantifying multiple sequence alignment uncertainties with GUIDANCE2 (Sela et al., 2015). The remaining OG sequences that are longer than 100 aligned amino acids (AA) and present in all species were then used to reconstruction preliminary maximum likelihood (ML) trees using IQ-TREE version 1.6.12 (Nguyen et al., 2014). PhyloTreePruner was used to identify the maximally inclusive subtree with each taxon represented by one sequence (Kocot et al., 2013). The predicted AA sequences were aligned with MAFFT version 7 (Katoh and Standley, 2013). The nucleotide coding sequences (CDS) were then aligned by using PAL2NAL according to the corresponding AA alignment (Suyama et al., 2006).

### Phylogenetic and Coalescent Analyses

To reconstruct the phylogeny of Nolinoideae, we employed maximum likelihood (ML), Bayesian inference (BI), and coalescent-based methods. CDS and AA sequences of all OGs were concatenated using FASconCAT-G version 1.02 (Kück and Longo, 2014). ML analyses were performed using the parallel version of IQ-TREE. ModelFinder was used in IQ-TREE to select the best model with free-rate heterogeneity based on BIC and AICc scores (Kalyaanamoorthy et al., 2017). Branch support was evaluated with 1,000 ultrafast bootstraps and SH-like approximate likelihood ratio tests using 1,000 replicates (Guindon et al., 2010; Hoang et al., 2017). In the BI analysis, parameters were set to aamodelpr = mixed and rates = gamma for AA sequences and nst = 6 and rates = gamma for nuclear sequences. Four chains were run for 1,000,000 generations and sampled every 1,000 generations with the first ca. 15% of the samples discarded as burn-in.

We used ASTRAL-III version 5.6.3 (Zhang et al., 2018), a quartet-based method under the multispecies coalescent, to estimate the species tree from nuclear gene trees. Each nuclear gene tree was generated with IQ-TREE, including 100 bootstrap replicates using the same parameter setting as above. Supports on the ASTRAL phylogeny were assessed using multi-locus bootstrap with 100 times of the gene tree bootstrap phylogenies and the more recently developed local posterior probability method, which estimates relative quartet support on each branch. In order to reflect uncertainty in gene tree estimates, another ASTRAL analysis was performed using the gene tree nodes collapsed with <50% bootstrap support (BS) with SumTrees.py from DendroPy 4.4.0 (Sukumaran and Holder, 2010).

Because of the debate of *Maianthemum* being close to *Polygonatum* group and the phylogenetic uncertainty of *Theropogon* within the subfamily, an approximately unbiased (AU) test (Shimodaira, 2002) was conducted to test alternative placements of each of them. The AU test compares log-likelihood scores among alternative trees using the CONSEL version 0.20 (Shimodaira and Hasegawa, 2001).

### Analyses of Conflicts

The presence of gene tree conflicts and concordance in the pseudocoalescence analyses was checked using PhyParts (Smith et al., 2015). This method allowed us to assess how many genes support or conflict with individual bipartitions within a species tree: if there is a dominant tree topology in the gene trees or, if there is conflict, whether this stems from an alternative tree topology, or from low-frequency alternative gene topologies, or lack of support for conflicting bipartitions. Gene trees used as input for ASTRAL and the resulting species tree generated by the program were rooted to be used as input in PhyParts, which was done using the program pxrr in the package phyx (Brown et al., 2017). Species trees were rooted having *Eriospermum* as outgroup. We ran PhyParts with the -b option set to 50 so that branches with less than 50% BS in the gene trees would not be considered. The results from PhyParts were used as input in the phypartspiecharts.py script^3^, to generate a species tree with pie charts in each node showing the proportion of concordant gene trees and conflicting topologies.

## Results

**FIGURE 1:**
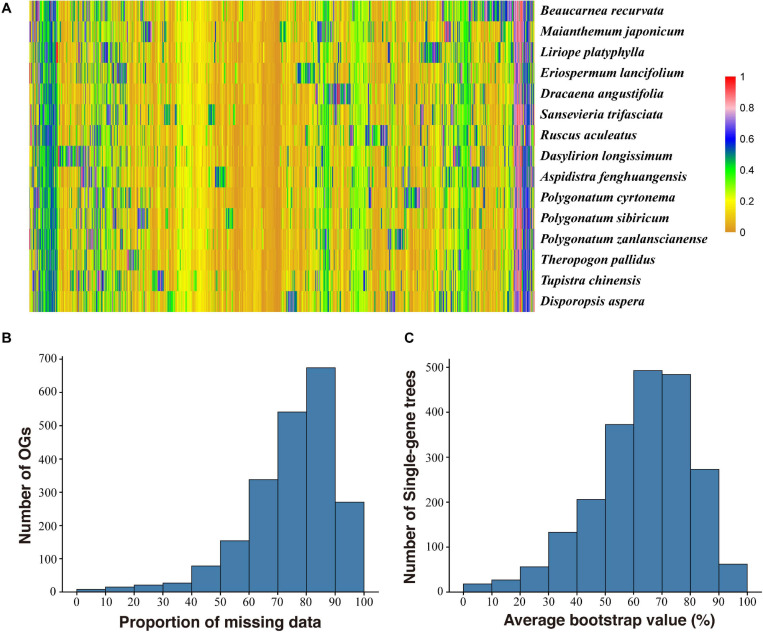
Frequency of missing data of each orthogroup (OG) in each taxon **(A)**, distribution of missing data in the OGs **(B)**, and average bootstrap value of single gene tree **(C)**. Each row corresponds to a taxon and each column corresponds to an OG in **(A)**.

A summary of the assembly statistics of the 15 transcriptomic data is shown in Table 1. After ORF prediction and redundancy reduction, 35,232–58,558 unigenes were retained. We obtained 2,126 putative one-to-one orthogroups (each taxon has only one sequence) after further stringent filtering. The mean proportion of missing data was 2.06% (0–20.77% in each OG) (Figure 1). The aligned length of the 2,126 concatenated AA sequences was 865,803 bp with 257,048 variable sites and 90,466 parsimony informative sites, and the aligned CDS sequences contain 2,850,331 bp with 911,833 variable sites and 369,821 parsimony informative sites (Table 2).

**TABLE 2:** Sequence information of AA and CDS of 2,126 unigenes.

	AA	CDS
Length of OGs (mean) (bp)	101–3,692	303–11,076
	(409.94)	(1,349.59)
Length of concatenated matrix (bp)	865,803	2,850,331
GC content	–	47.4%
No. of variable sites	257,048	911,833
No. of parsimony informative sites (percentage)	90,466 (10.45%)	369,821 (12.97%)
No. of singletons	164,265	541,911

**FIGURE 2:**
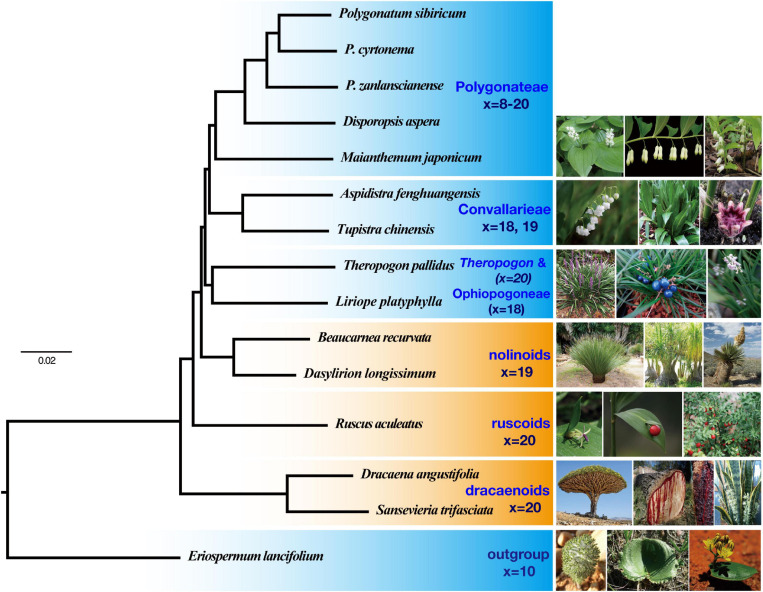
A partitioned maximum likelihood tree of Nolinoideae reconstructed from the concatenated alignment of 2,126 coding sequences. All branches are supported by 100% bootstrap values and posterior probability of 1.00.

Both the concatenation based on CDS and AA matrices yielded similar topologies (Figure 2), with all nodes supported by 100% BS values and posterior probability (PP) of 1.00, respectively (Supplementary Figures 1–3). The sister relationship between *Theropogon* and Ophiopogoneae has been robustly supported with BS = 100% and PP = 1.00, but with weak SH-like supports in both AA and CDS datasets (Supplementary Figures 1, 2). The coalescent analyses from ASTRAL produced similar topologies to those of concatenation results with difference from the placement of *Theropogon* (Supplementary Figures 4, 5). A total of 760 OGs were produced using the BUSCO approach. The ML tree of the concatenated sequences and the species tree (Supplementary Figures 6, 7) are similar to those of the large dataset with 2,126 OGs.

**FIGURE 3:**
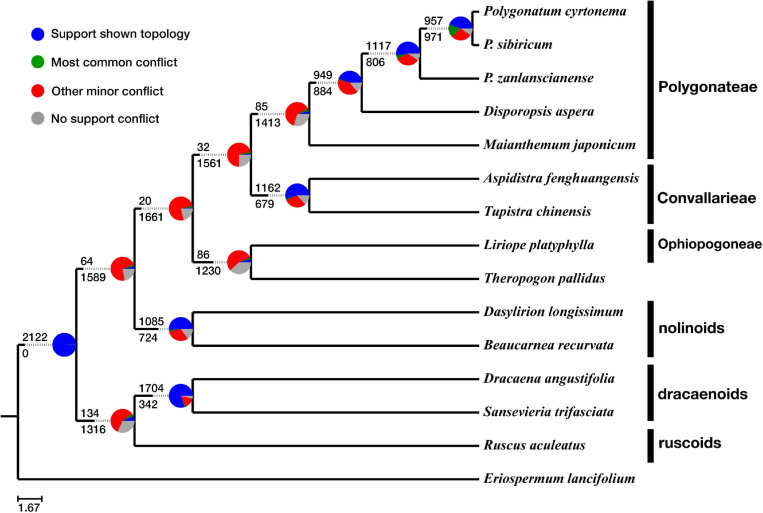
ASTRAL-III tree shows gene–tree conflict with pie chart at each node as the following: proportion of gene trees in concordance (blue), in conflict with the dominant alternative topology (green), in conflict with all other topologies (red), and unsupported with less than 50% bootstrap scores (gray).

**FIGURE 4:**
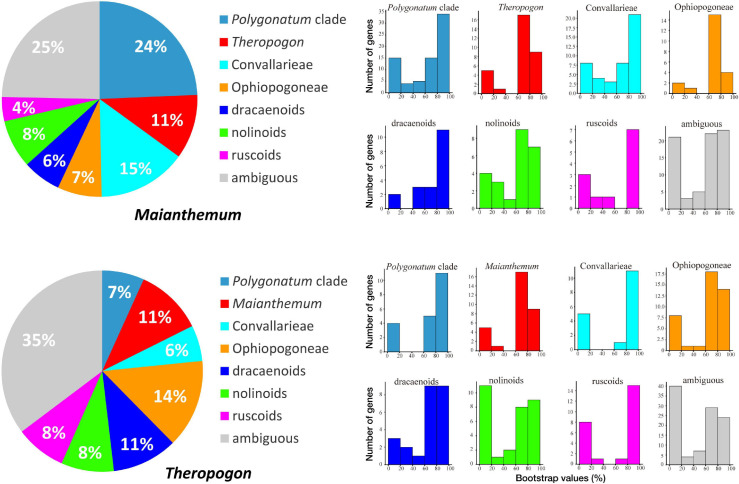
Numbers of individual gene trees supporting different relationships. Pie graphs show the proportions of gene trees with different topologies. Histograms indicate distributions of maximum likelihood (ML) bootstrap supports for the corresponding topologies.

In our phylogenetic results, the dracaenoid clade (represented by *Sansevieria* and *Dracaena*) is supported to be sister to a clade including the ruscoids further sister to a clade including the nolinoids and the herbaceous Convallariaceae clade (Figure 2). In the second ASTRAL species tree, the ruscoids are sister to the dracaenoids (Figure 3). Within the traditional Convallariaceae group, Ophiopogoneae + *Theropogon* were recovered as the first lineage sister to Convallarieae + Polygonateae. Based on the AU test, *Maianthemum* sister to *Polygonatum* group and *Theropogon* sister to the Ophiopogoneae were the best topologies, and other topologies about the phylogenetic placements of *Maianthemum* and *Theropogon* were rejected (*p*-values < 0.005). The best topologies suggested by the AU test were also supported by more single-gene trees (Figure 4). After stripping out the single-gene trees with low average support (BP < 50%) to reduce topological uncertainty, 24% and 14% of 785 gene trees supported a sister relationship between *Maianthemum* and *Polygonatum* clade and *Theropogon* as sister to the Ophiopogoneae, respectively (Figure 4).

Conflicts of gene trees against species tree are shown in Figure 3. A high level of concordance (>50%) was observed for the monophyly of Convallarieae, the nolinoids, and the dracaenoids, respectively. In the ASTRAL tree, the only well-supported clade in the gene trees was the node next to the root (the Nolinoideae excluding *Eriospermum* lineage), supported by 2,122 (∼99.8%) of the 2,126 loci tree topologies, and only 4 (0.2%) of the gene trees have no supported conflicts (Figure 3). We found only 134 of the 2,126 gene trees concordant with the species tree surrounding the sister relationships between the ruscoids and dracaenoids. The monophyly of the major lineages was supported by different numbers of gene trees (Figure 4). More than 80% of gene trees supported the monophyly of the dracaenoids and less gene trees (ca. 50%) supported the monophyly of the nolinoids and tribe Convallarieae, respectively (Figure 3). The sister relationship between *Maianthemum* and *Polygonatum* lineages was only supported by 85 gene trees (4.0%). The same pattern was observed in the node linking the *Theropogon* and the tribe *Ophiopogoneae* (Figure 3).

## Discussion

### Deep Relationships Within the Subfamily

Previous phylogenetic works have greatly improved our understanding of the relationships of Nolinoideae (Rudall et al., 2000; Jang and Pfosser, 2002; Kim et al., 2010), but robust resolution of their early divergences has proven to be a formidable task. Here, with our transcriptomic dataset, we reconstructed most relationships with strong supports (Figure 2). The higher supports were found for deeper-level relationships that previously had weak supports (e.g., Polygonateae and Convallarieae), as well as for new hypotheses (e.g., *Theropogon* as sister to Ophiopogoneae).

The tree topology of Nolinoideae reconstructed in this study was largely congruent with previous results (Rudall et al., 2000; Jang and Pfosser, 2002; Kim et al., 2010). Regardless of which datasets were analyzed and what methods were utilized, all phylogenetic analyses consistently support the dividing of whole Nolinoideae into seven major lineages: (1) *Eriospermum*, (2) dracaenoids, (3) ruscoids, (4) nolinoids, (5) Ophiopogoneae, (6) Convallarieae, and (7) Polygonateae (Figures 2, 3). In addition, the uncertain *Theropogon* is suggested to be close to Ophiopogoneae. These seven groups were also recognized by the previous works based on both molecular and/or morphological evidences (Rudall et al., 2000; Yamashita and Tamura, 2000b; Pires et al., 2006; Kim et al., 2010). In particular, most nodes in this transcriptome-based phylogeny are robustly supported by BS = 100% and PP of 1.00 (Figure 2), in contrast to previous studies that most internal relationships among these seven clades have extremely low supports (Rudall et al., 2000; Jang and Pfosser, 2002; Kim et al., 2010; Seberg et al., 2012). Furthermore, except for the *Eriospermum* lineage as outgroup, transcriptomic results could largely classify the remaining six lineages into two groups, one is a paraphyletic grade predominantly characterized by woody habits and a monophyletic group, previously recognized as the Convallariaceae, uniformly characterized by herbaceous life forms (Figure 2).

The three woody lineages of dracaenoids, ruscoids, and nolinoids are not sister related but form a paraphyletic grade with the dracaenoids sister to the ruscoids and then the nolinoids (Figure 2). These woody taxa share the tenuinucellate parietal cells and the same basic number of *x* = 19–20 (Rudall et al., 2000).

The dracaenoids have a complex taxonomic history and their taxonomic placement has been changed several times, including classification in Liliaceae (Brown, 1915), Agavaceae (Cronquist, 1981; Bogler and Simpson, 1996), Dracaenaceae (Dahlgren et al., 1985), Ruscaceae (Chase et al., 1995), Convallariaceae (Yamashita and Tamura, 2000b), and, finally, Asparagaceae subfamily Nolinoideae (APGIII, 2009). Within Nolinoideae, the phylogenetic relationship of the dracaenoids is still unresolved. The dracaenoids are differed from the nolinoids in having berries, no oils in guard cells, and mucilage-filled cells with crystal raphides. Some previous studies have suggested the dracaenoids have a close relationships with the nolinoids, but with very low supports (Rudall et al., 1997; Kim et al., 2010). Our results suggested that the dracaenoids have a closer relationship with the ruscoids than the nolinoids (Figure 2). Chromosome numbers are probably more similar between the dracaenoids (*x* = 20) and the ruscoids (*x* = 20) than between the dracaenoids and the nolinoids (*x* = 19). Interestingly, the ruscoids were suggested to be directly sister to the dracaenoids in the second ASTRAL species tree (Figure 3).

The ruscoids have once been considered the most closely related to the traditional Asparagaceae by many botanists (Dahlgren et al., 1985; Takhtajan, 1997). However, analysis of molecular sequence data indicated a close relationship with Convallariaceae taxa (Chase et al., 1995). Karyotypes are probably more similar between ruscoids (*x* = 20) and some Convallariaceae s.l. (mainly *x* = 18, 19) than between Ruscaceae and *Asparagus* (*x* = 10) (Tamura, 1995; Rudall et al., 2000). Ruscoids lack phytomelan in the seed coat, a relatively consistent apomorphy shared with Convallariaceae s.l., and are serologically closer to Convallariaceae s.l. than to *Asparagus* (Chupov and Cutjavina, 1980; Rudall and Campbell, 1999). Our study suggested a relatively basal position within the subfamily as the second woody lineages between the dracaenoids and the nolinoids (Figure 2).

The nolinoids were originally placed in the broadly defined and traditional family of Liliaceae and then in the tribe Dracaeneae or Nolinaceae (Rudall et al., 2000). All taxa from Nolinaceae, Dracaeneae, and Yuccoideae were placed in Agavaceae based on their fibrous leaves and anomalous woody growth of a secondary thickening meristem (Hutchinson, 1934). However, this placement was not supported by the other morphological evidence (flowers, fruits, and seeds) and the chromosome data (Sharma and Chaudhuri, 1964; Rudall et al., 2000). Several other studies proposed a close relationship to Convallariaceae and Dracaenaceae (Bogler et al., 1995; Bogler and Simpson, 1996), particularly Ophiopogoneae (Rudall et al., 2000; Yamashita and Tamura, 2000b), but there are no obvious morphological evidence to support this affinity. More recently, the nolinoids were suggested to be close to Convallariaceae–Dracaenaceae–Ruscaceae s.s. in the maximum parsimony analysis while close to the Aspidistreae–Convallarieae group in the Bayesian analysis (Kim et al., 2010). In the present study, the nolinoids are suggested to be closer to the herbaceous Convallariaceae group than other woody lineages (Figure 2). It is difficult to find any obvious synapomorphies of them, but chromosome data provide possible insights as the nolinoids usually with *x* = 19 and the Convallariaceae group dominantly featured with *x* = 18–19 (Figure 2).

The herbaceous clade, consists of three tribes sharing sympodially or monopodially branching rhizomes, is confirmed with strong BS and PP supports as revealed by some previous analyses (Rudall et al., 2000; Yamashita and Tamura, 2000b). Based on the phylogenomic tree, our data suggest Ophiopogoneae + *Theropogon* diverged first, sister to a clade that is composed of Convallarieae (including *Aspidistra* and *Tupistra*) and the monophyletic Polygonateae (Figure 2).

The Ophiopogoneae was treated as a well monotypic member of Convallarieae s.s., but its phylogenetic relationship to the other herbaceous tribes has long been in uncertainty within the traditional Convallariaceae (Rudall et al., 2000; Yamashita and Tamura, 2000b; Kim et al., 2010; Wang and Yang, 2018). In our analyses, Ophiopogoneae is represented by *Liriope platyphylla*, together with *Theropogon*, which is suggested to be sister to Convallarieae + Polygonateae (Figure 2). Based on comparative plastid genomic data, Floden and Schilling (2018) provided similar results and suggested that Ophiopogoneae was close to a clade including the Polygonateae and Aspidistreae (treated as Convallarieae).

Convallarieae share some synapomorphic characters such as the chromosome basic number (*x* = 19), usually berries, and non-septal nectaries (Dahlgren et al., 1985). Rudall et al. (2000) demonstrated close relationships between Convallarieae and the ruscoids, but the latter is distinguished with their basic number (*x* = 20) and septal nectaries from Convallarieae. The present study suggested Convallarieae is close to Polygonateae (Figure 2). Moreover, based on comparative plastid genomics, Floden and Schilling (2018) regarded *Aspidistra* from Convallarieae as a separate lineage close to Polygonateae.

### Phylogenetic Placement of *Maianthemum* and *Theropogon*

The monophyly of Polygonateae with two lineages has been recovered by our transcriptome data, similar to most other molecular studies (Rudall et al., 2000; Yamashita and Tamura, 2000b; Meng et al., 2008, 2014). However, a few other phylogenetic studies have recovered the non-monophyly of the tribe, such as in the plastid matK + rbcL and rDNA 18S ML tree (Kim et al., 2010) and a combined six-gene matrix (Seberg et al., 2012). A more recent study based on the whole plastid genomes suggested that the terminal-flowered *Maianthemum* is sister to Ophiopogoneae other than the other members of the Polygonateae (Floden and Schilling, 2018). Here, we tested the phylogenetic status of *Maianthemum* using topological statistics from single-gene trees. Except for the uncertainty, the results indicate that the largest number of single-gene trees (24%) supports the close relationship with *Polygonatum* lineage (Figure 4). Only 11% single-gene trees support the close relationship between *Maianthemum* and Ophiopogoneae (Figure 4).

The large dataset generated in our study provide a unique insight into the sources of this topological instability, especially evaluating phylogenetic placement of *Theropogon*. It was treated as a member of Convallarieae by morphological characters (Vaikos et al., 1989). However, all previous analyses of molecular data have failed to support the placement of *Theropogon* in Convallarieae clade or herbaceous lineage, but always showing close relationship to the ruscoids or dracaenoids, all of which also shared similar basic chromosome numbers (*x* = 20) (Rudall et al., 2000; Yamashita and Tamura, 2000a, b). Our transcriptome data based on concatenated ML, BI, and one ASTRAL analysis indicate that it is sister to Ophiopogoneae (Figures 2, 3), but collapsed in the other coalescent ASTRAL analyses (Supplementary Figures 4, 5). Except for the uncertainty, the highest number of single-gene trees (14%) supports a close relationship with Ophiopogoneae (Figure 4). Our results suggested a possible reticulate evolution had occurred in the early origin of *Theropogon* as evidenced by the complicated distributions of single-gene trees (Figure 4). The discordance could result from horizontal gene transfer, incomplete lineage sorting, and/or ancient hybridization between different ancestral lineages.

### Concordance and Conflicts Among Gene Trees

Even though the backbone nodes showed BS = 100% and PP = 1.00 in the super-matrix approach (Figure 2), the PhyParts analysis on the ASTRAL species tree showed a high degree of gene–tree conflicts (Figure 3). High gene–tree conflicts (>75%) were prevalent across many relationships including the position of the nolinoids, the relationship of *Theropogon pallidus* and Ophiopogoneae, the relationship of the Convallarieae and the Polygonateae, and the relationship of the genus *Maianthemum* with respect to the *Polygonatum* and *Disporopsis* (Figure 3).

Only 1.5% gene trees support and dominance of other minor conflicting bipartitions (73.4%) were found for the sister relationship between Polygonateae and Convallarieae. The nolinoids as sister to the herbaceous group was supported only by 64 (3.0%) gene trees, and the ruscoids as sister to the dracaenoids by 134 (6.3%) gene trees (Figure 3). Low gene tree supports and dominance of other conflicting bipartitions were also detected in the other clades. Similar patterns were observed for grouping the Ophiopogoneae–*Theropogon* and Convallarieae–Polygonateae in a single clade (Figure 3). Although a very low number of gene trees supported the deeper relationships within the subfamily, there was no dominant alternative topology found among the conflicting topologies (indicated in green in the pie charts of Figure 3), but they exhibited the dominance for other minor conflicting topologies as indicated in red in the pie charts of Figure 3.

Similar to other studies on plants and animals with multi-locus datasets, it seems to be unnecessary that the analyses of multiple gene copies have to be in concordance or high support of the topologies resulted from coalescent-based methods of species tree estimations (Jeffroy et al., 2006; Sun et al., 2015; Tang et al., 2015; Siniscalchi et al., 2019). On the other hand, individual gene trees with various topologies are commonly found in many taxa on different levels, suggesting that incomplete lineage sorting, hybridization, gene duplication, and horizontal gene transfer are pervasive phenomena and could be significant causes of these topological conflicts or discordances (Yu et al., 2013; Sun et al., 2015; Tang et al., 2015; Mallo and Posada, 2016; Arcila et al., 2017; Bogarín et al., 2018).

Incomplete lineage sorting might cause strong conflicts in taxa with closely related species with fast diversifications due to the alleles within a population without enough time to coalesce (Degnan and Rosenberg, 2009; Meyer et al., 2016; Arcila et al., 2017). Except for incomplete lineage sorting and early speciation, discordance could also be resulted from estimation mistakes in the sequence alignments of individual genes, such as missing sequences, phylogenetic noise, or long-branch attractions (Mallo and Posada, 2016; Mirarab et al., 2016). Including gene alignments without missing sequences did obtain some difference in topologies, but the major differences are mostly from the position of some taxa, such as *Theropogon* and *Maianthemum* as shown in Figure 4. Additional investigation with plastid genomic data and comprehensive analyses of both nuclear and plastid data are required to clarify the complex evolution history within the subfamily.

## Conclusion

The utility of transcriptome phylogenetics is demonstrated by the reconstruction of the relationships within Nolinoideae. Rapid diversification or complex evolution is an obstacle to well-resolved relationships, which can be remedied with increased sequence data (Xi et al., 2012; Wen et al., 2015b). Relationships within the subfamily have long been obscured by a rapid diversification and now the phylogeny is becoming more resolved. The three woody lineages of dracaenoids, ruscoids, and nolinoids are not sister relationship but are forming a paraphyletic grade with dracaenoids as the first diverged lineage followed with ruscoids and then nolinoids (Figure 2). The monophyly of herbaceous clade is confirmed with strong BS, which consists of three tribes sharing sympodially or monopodially branching rhizomes (Rudall et al., 2000; Yamashita and Tamura, 2000b). Our study further revealed a low level of gene trees supported the backbone relationships within the Nolinoideae and most of the conflicts were located at deeper nodes along the phylogeny (Figure 3), indicating that incomplete lineage sorting, hybridization, and gene duplication are all possible causes of these topological incongruences. Further works are necessary to uncover their complex evolution using more advanced genomic data such as genomic skimming or target enrichment technologies (Mamanova et al., 2010; Wen et al., 2015b). This work paves the way for investigations on evolutionary history of the highly morphological heterogeneous Nolinoideae on the genomic level.

## Data Availability Statement

Raw data in the fastq format are deposited at the NCBI Sequence Read Archive, under BioProjects PRJNA608213, and other data can be found at https://data.mendeley.com/datasets/ncy4td2hb9.

## Author Contributions

RM designed the study, collected samples, conducted lab work, conducted data analyses, and wrote the manuscript. L-YL and D-GZ participated in field collections and provided samples. YM and Z-LN helped to design the study, wrote the Discussion section, and reviewed and commented on the manuscript. J-YZ provided samples, collaborated on lab work, conducted data analyses, and reviewed and commented on the manuscript. All authors contributed to the article and approved the submitted version.

## Conflict of Interest

The authors declare that the research was conducted in the absence of any commercial or financial relationships that could be construed as a potential conflict of interest.

## Footnotes

1 http://www.bioinformatics.babraham.ac.uk/projects/fastqc

2 https://github.com/TransDecoder/TransDecoder/releases

3 https://github.com/mossmatters/phyloscripts/tree/master/phypartspiecharts
