# Supplementary material for: The Deep Evolutionary Relationships of the Morphologically Heterogeneous Nolinoideae (Asparagaceae) Revealed by Transcriptome Data
Source: Front Plant Sci. 2021 Jan 14;11:584981. doi: 10.3389/fpls.2020.584981 (PMC7840527; doi:10.3389/fpls.2020.584981)
Supplement: Supplementary file 1 [file Data_Sheet_1.PDF]

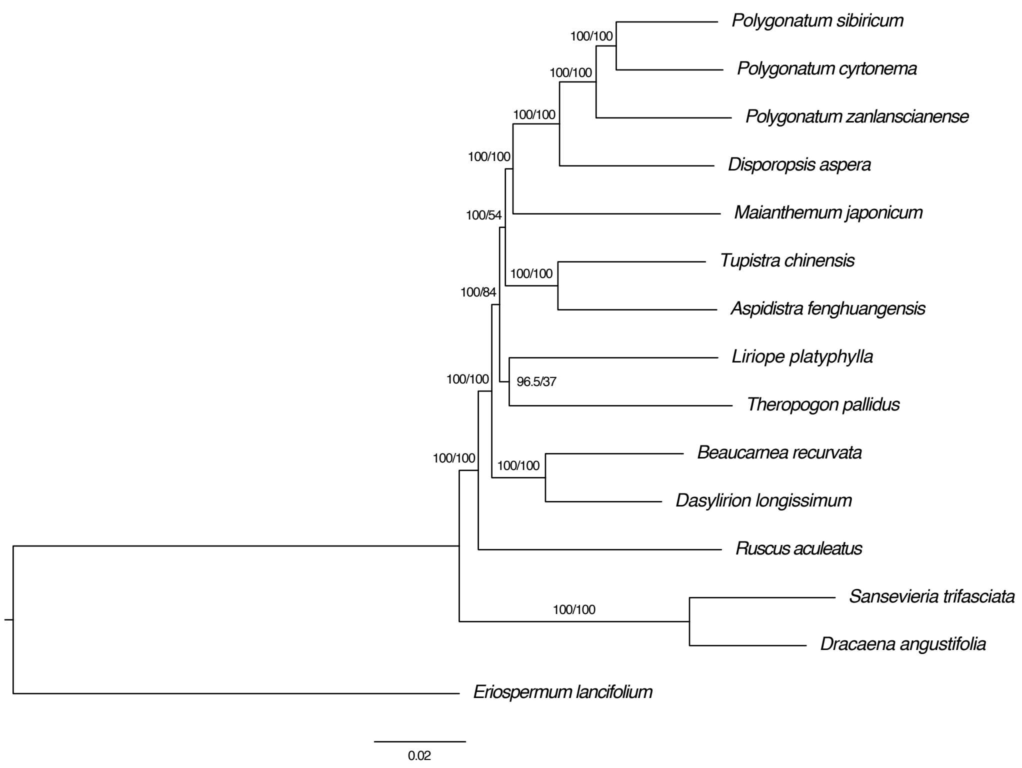

Fig. S1 A partitioned ML tree of subfamily Nolinoideae reconstructed from the concatenated alignment of CDS of 2126 OGs. The bootstrap values are shown before dash lines and SH-like supports after dash lines.

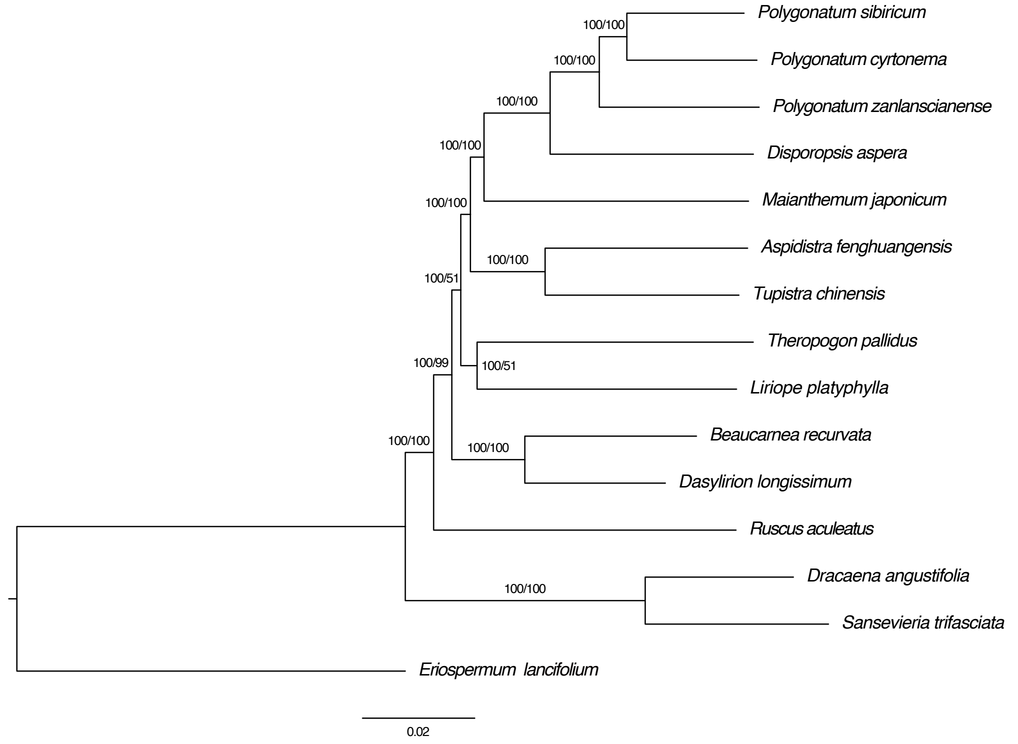

Fig. S2 A partitioned ML tree of subfamily Nolinoideae reconstructed from the concatenated alignment of AA of 2126 OGs. The bootstrap values are shown before dash lines and SH-like supports after dash lines.

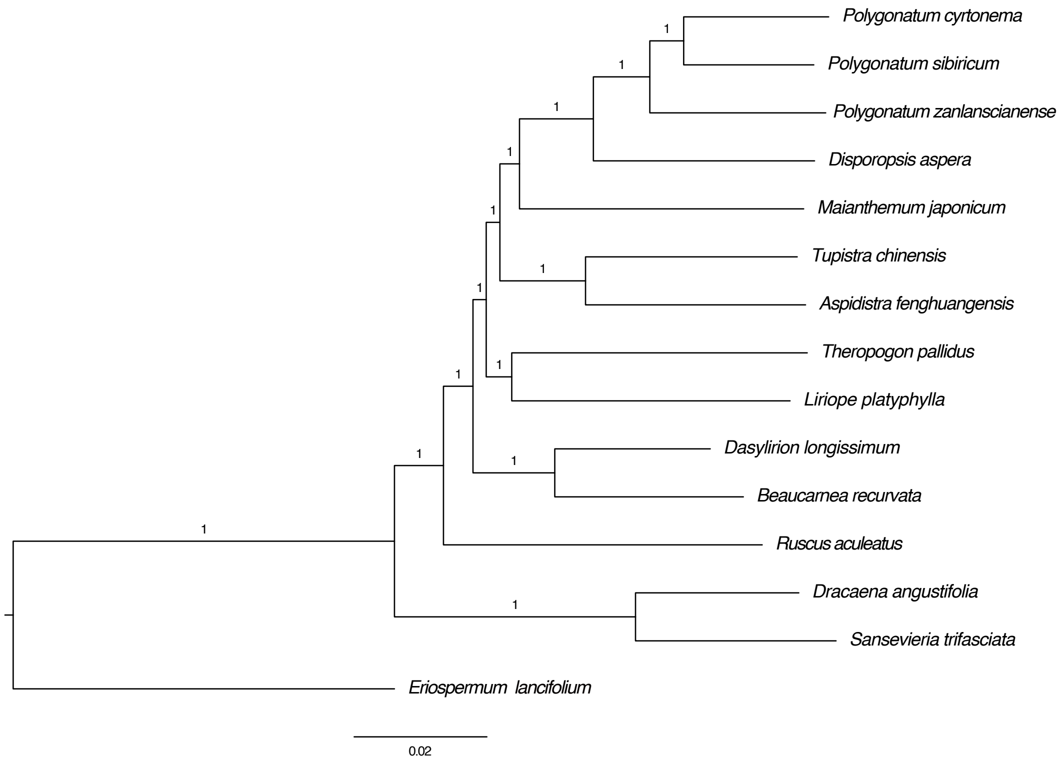

Fig. S3 A Bayesian tree of subfamily Nolinoideae reconstructed from the concatenated alignment of CDS of 2126 OGs. All branches are supported by posterior probability of 1.00.

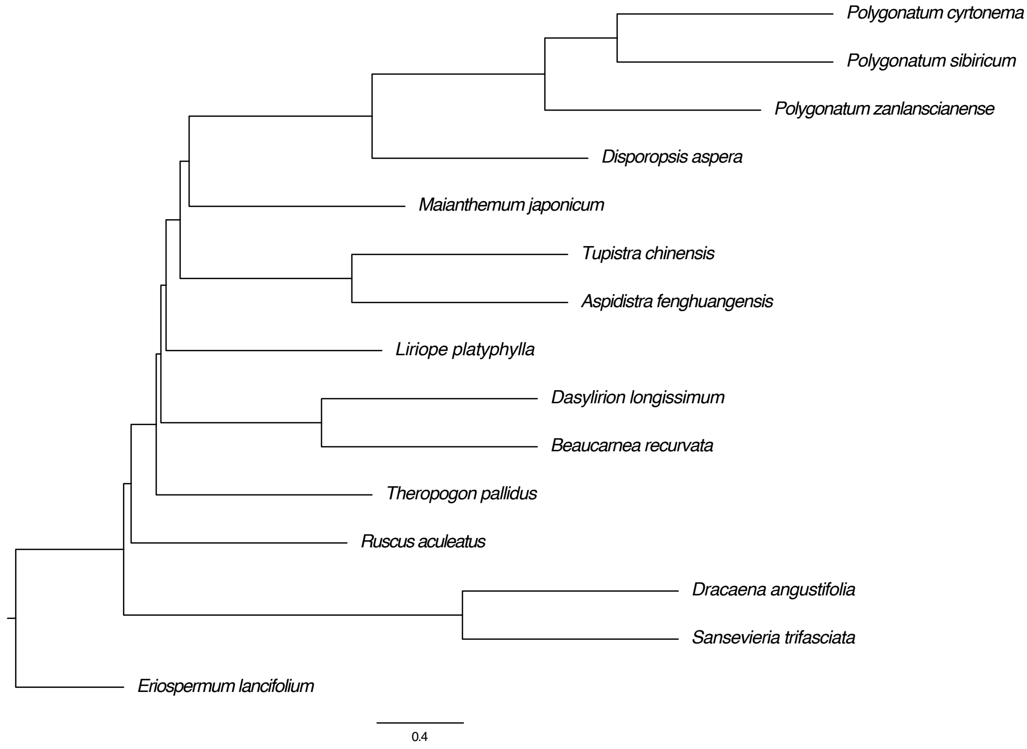

Fig. S4 Species tree of Nolinoideae reconstructed from ASTRAL-III based on CDS gene trees of 2126 OGs.

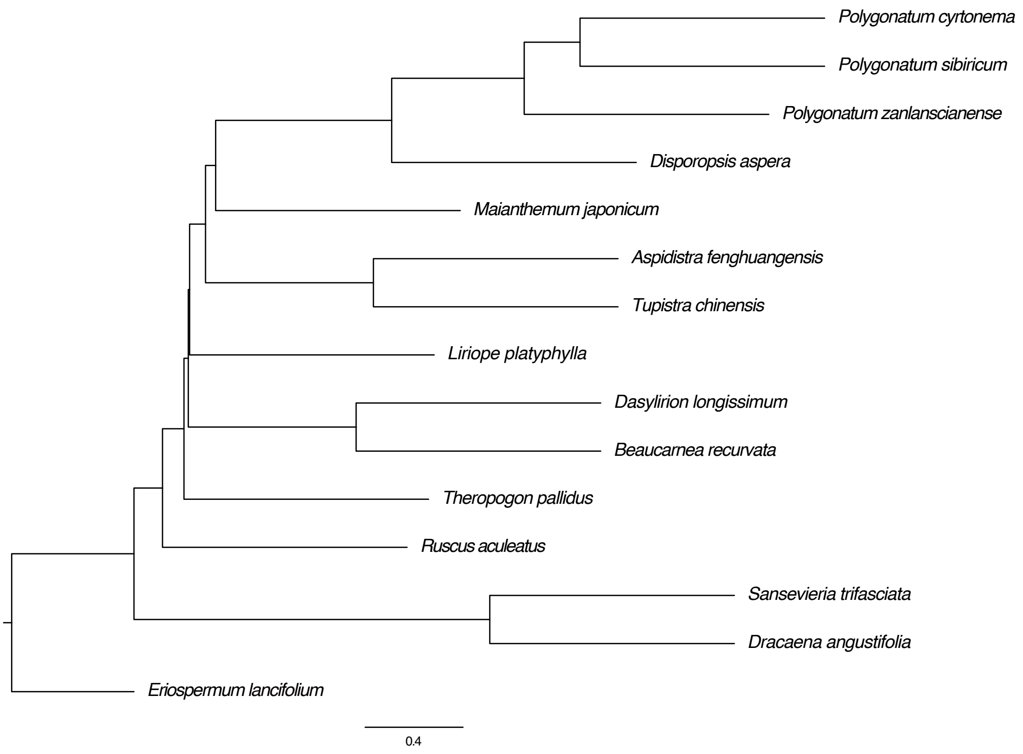

Fig. S5 Species tree of Nolinoideae reconstructed from ASTRAL-III based on AA gene trees of 2126 OGs.

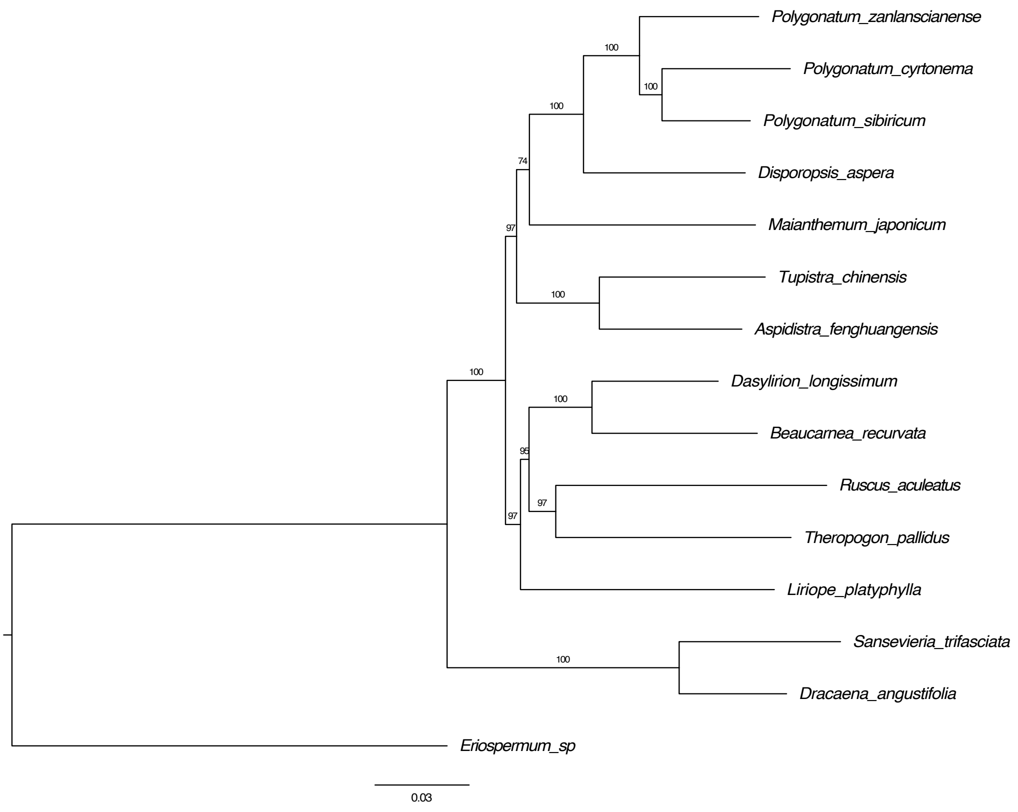

Fig. S6 A partitioned ML tree of subfamily Nolinoideae reconstructed from the concatenated alignment of AA of 760 OGs. The bootstrap values are shown above branches.

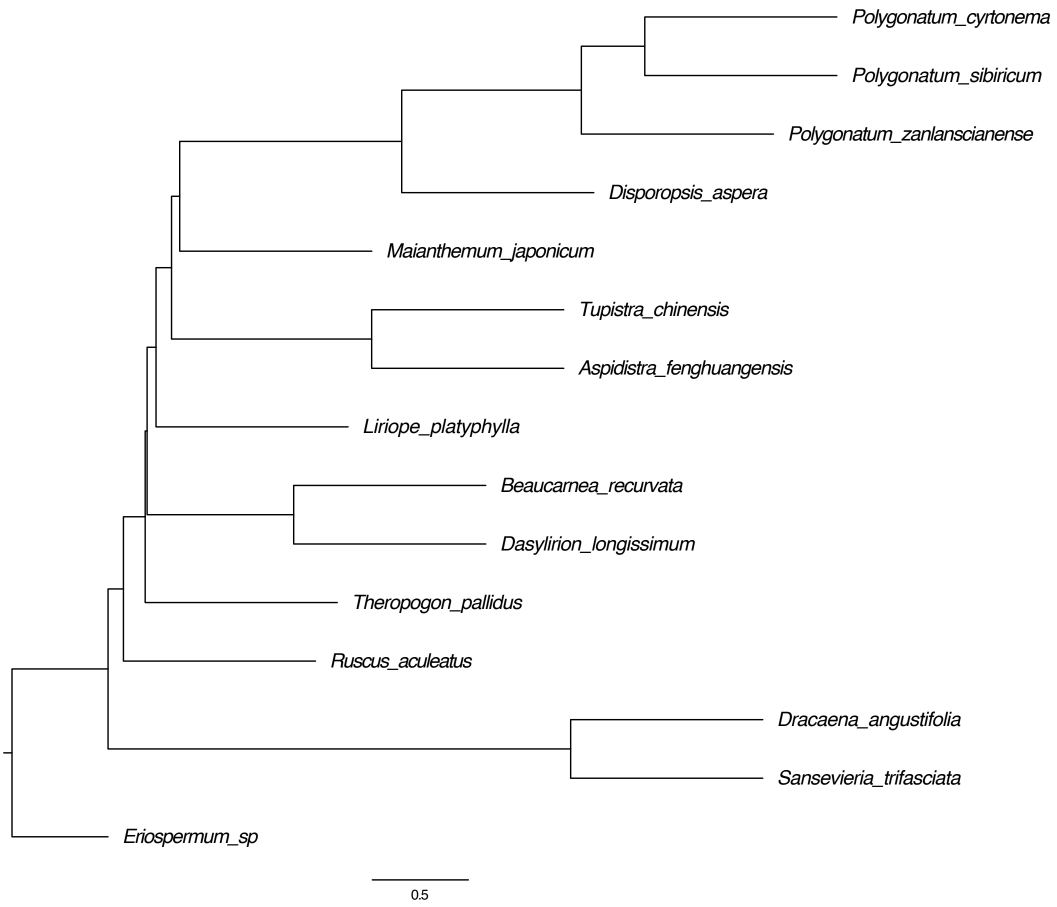

Fig. S7 Species tree of Nolinoideae reconstructed from ASTRAL-III based on AA gene trees of 760 OGs.
